# Generation and Characterization of Anti-CD34 Monoclonal Antibodies that React with Hematopoietic Stem Cells

**Published:** 2014-10-04

**Authors:** Leili Aghebati Maleki, Jafar Majidi, Behzad Baradaran, Aliakbar Movassaghpour, Jalal Abdolalizadeh

**Affiliations:** 1 Immunology Research Center, Tabriz University of Medical Sciences, Tabriz, Iran; 2 Department of Immunology, Faculty of Medicine, Tabriz University of Medical Sciences, Tabriz, Iran

**Keywords:** Monoclonal Antibody, CD34, Hematopoietic Stem Cells, Isolation

## Abstract

CD34 is a type I membrane protein with a molecular mass of approximately 110 kDa. This antigen is associated with human hematopoietic progenitor cells and is a differentiation stage-specific leukocyte antigen. In this study we have generated and characterized monoclonal antibodies (mAbs) directed against a CD34 marker. Mice were immunized with two keyhole lympet hemocyanin (KLH)-conjugated CD34 peptides. Fused cells were grown in hypoxanthine, aminopterine and thymidine (HAT) selective medium and cloned by the limiting dilution (L.D) method. Several monoclones were isolated by three rounds of limited dilutions. From these, we chose stable clones that presented sustained antibody production for subsequent characterization. Antibodies were tested for their reactivity and specificity to recognize the CD34 peptides and further screened by enzyme-linked immunosorbent assay (ELISA) and Western blotting analyses. One of the mAbs (3D5) was strongly reactive against the CD34 peptide and with native CD34 from human umbilical cord blood cells (UCB) in ELISA and Western blotting analyses. The results have shown that this antibody is highly specific and functional in biomedical applications such as ELISA and Western blot assays. This monoclonal antibodies (mAb) can be a useful tool for isolation and purification of human hematopoietic stem cells (HSCs).

The CD34 antigen is a single chain transmembrane glycoprotein with a molecular weight of approximately 110 kDa that is expressed on human hematopoietic progenitor cells, embryonic fibroblasts and some cells in fetal and adult nervous tissue (1). CD34 molecule density is highest on early hematopoietic progenitor cells and decreases as cells mature (2). Full-length and truncated forms of CD34 exist-both are expressed on the cell surface (3, 4). Anti-CD34 can be used to identify CD34 expression in a variety of neoplasias including vascular tumors and acute lymphoblastic leukemias (5). This molecule, initially identified as an antigen expressed on hematopoietic stem cells (HSCs) has since been widely used as a marker for the isolation of hematopoietic cells (6).

The purpose of this study was to produce and characterize monoclonal antibodies (mAbs) specific for the CD34 antigen by immunizing Balb/c mice with two synthetic peptides derived from the extracellular domain of human CD34 in order to develop a diagnostic tool for detection and isolation of HSCs.

For this study, the amino acid sequence of human CD34 was carefully analyzed. Then, two 14-mer synthetic peptides that sequenced TFSNVSTNVSYQET and NTNSSVQSQTSVIS from the extracellular portion of the human CD34 protein were designed and selected as the immunogen, based on local hydrophilicity as predicted by the method of Hopp and Woods (7). The designed peptide was separately conjugated to keyhole lympet hemocyanin (KLH) and bovine serum albumin (BSA) (Thermo, USA), following the procedure provided by the manufacturer (8). At the next step we used four, 6-week-old Balb/c female mice for peptide immunization. All animal experiments in this research followed the guidelines of the Laboratory Animal Ethical Commission of Tabriz University of Medical Sciences. Each mouse was immunized 4 times over a 2-3 week interval. One week after the last immunization, blood was taken from each mouse by a vertical incision of the tail vein (after anesthesia with ether for pain prevention) and the antibody response was measured by enzymelinked immunosorbent assay (ELISA) as described previously (9). The mouse with the highest serum antibody titer was selected as the spleen donor. Next, to collect mouse peritoneal macrophages as a feeder layer, we injected RPMI-1640 media into the peritoneal cavity of an unimmunized Balb/c mouse followed by subsequent aspiration and collection of the peritoneal cells. Mouse myeloma SP2/0 cell line was used as the fusion partner. Thus, one week before fusion cells were cultured in RPMI (Gibco) and 10% FBS until they attained >70% confluency in the logarithmic phase. The spleen cells of the immune mouse were removed under sterile conditions. Spleen cells were fused with SP2/0 cells at a 5 to 1 ratio by PEG1450 (Sigma-Aldrich Co. St. Louis, MO, USA) as the fusogen. Supernatants of the growing wells were screened for the production of antibody using an ELISA method as described previously (9).

After screening, clones that had high absorbance were selected for cloning by the limiting dilution (L.D) method. Suitable monoclones that possessed high absorbance were selected for characterization of antibodies. The class and subclass of mAbs were determined by an ELISA with a mouse monoclonal subisotyping kit that contained rabbit anti-mouse IgG1, IgG2a, IgG2b, IgG3, IgM and IgA following the procedure provided by the manufacturer (Thermo, USA) (9). At the next step, the mAbs were purified from culture supernatants using sepharose beads conjugated with protein A column affinity chromatography according to isotype. Confirmation of the mAb purity was monitored by SDS-PAGE under a non-reducing condition.

We next attempted to determine whether these antibodies were capable of identification and ultimate enrichment for hematopoietic stem/progenitor cells. To accomplish this, we used Western blotting according to the protocol we described elsewhere with minor modification (10). Initially, umbilical cord samples were obtained from the umbilical vein after the vaginal delivery of normal-term babies following informed consent by the healthy mothers. Then, mononuclear cells (MNC) were isolated by ficoll-hypaque (1.077 g/ml, Pharmacia Biotech) density gradient centrifugation (10). Next, we choose a panel of different cell lines with origin of blood such as Raji and HL-60 for the cross-reactivity assay. In addition, cells were cultured in their recommended medium, harvested and lysed with lysis buffer. The protein concentration of lysate was measured by a biophotometre (Ependorff, Germany). The samples were loaded onto a 12.5% SDS-PAGE gel at 100 V for 2 hours. After electrophoresis, the SDS-PAGE gel was transferred electrophoretically to wet nitrocellulose membrane. Transfer of proteins from the gel to a nitrocellulose membrane was undertaken at 100 mA for 2 hours. Then, the membrane was developed using an enhanced chemiluminescence detection system (ECL, Amersham Phamacia Biotech Inc., USA).

In this study, we used the peptide-KLH for mice immunization and peptide-BSA for conjugation assessment and specificity testing of the antibody. Due to the very high molecular weight of KLH, it is not possible to run the KLH conjugate on SDS-PAGE. In this context, BSA conjugate was used for efficacy of conjugation. The coupling efficiency, as determined by the SDS-PAGE peptide was suitable. Four mice were immunized four times against KLH-conjugated peptides. Then, we evaluated sera from the mice in direct binding assays for antibody reactivity with BSA-peptide conjugate. Serum of the immune mouse at the 1:8000 dilution displayed a high absorbance in reaction with BSA-peptide by indirect ELISA (Fig 1). Accordingly, the mouse with a higher titer of specific antibody (Mouse 3) was selected for hybridoma production. Spleen cells from the immune mouse were fused with myeloma SP2/0 as the fusion partner (Fig 2A). The fused cells were suspended in hypoxanthine, aminopterine and thymidine (HAT) medium and distributed into five culture plates that contained feeder layer (Fig 2B).

Several anti-CD34 mAbs that produced hybridomas were obtained. From these, the 3D5 clone showed high reactivity with immunogenic peptide in the ELISA assay (Figs 2C, D). For this reason, we performed all subsequent tests with this clone. Further characterization of this antibody showed that it was an IgG1 isotype with a kappa light chain (Table 1). We assessed purity of this antibody by SDS-PAGE. A single band of approximately 150 kDa in SDS-PAGE analysis indicated the proper purification of the antibody (Fig 3A).

**Fig 1:**
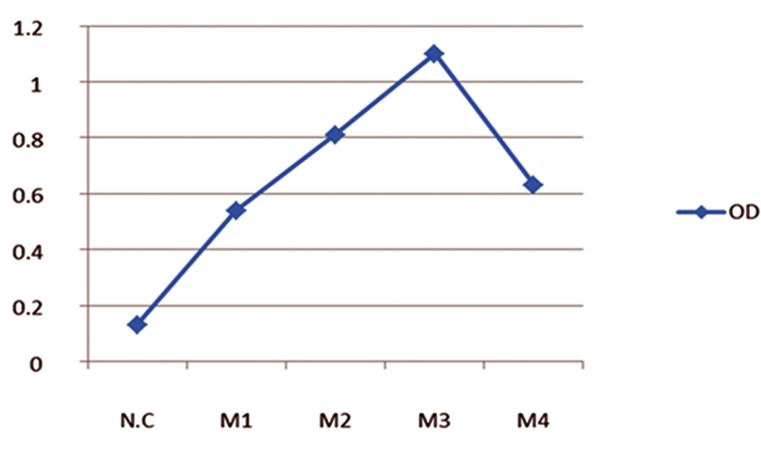
Evaluation of anti-CD34 antibody production with immunizing peptide in the serum of immunized mice by ELISA. The serum of the immune mouse and the non-immune mouse negative control were diluted 1:8000. NC; Negative control, M1; Mouse 1, M2; Mouse 2, M3; Mouse 3, M4; Mouse 4 and OD; Optical density.

**Fig 2:**
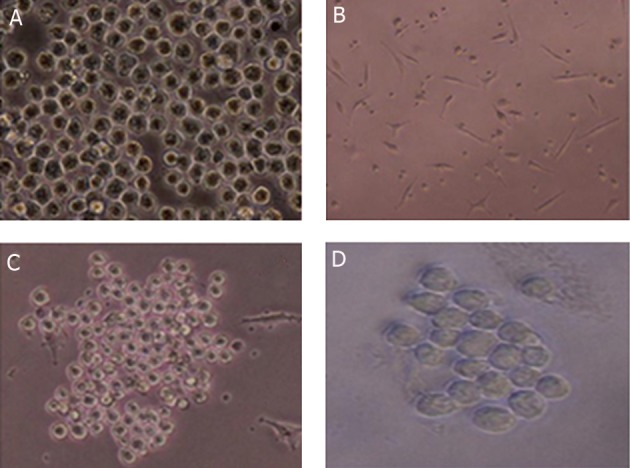
Proliferated monoclone with higher optical density as the suitable monoclone. A. Sp2/0 in logarithmic phase.), B. Mouse peritoneal macrophages as feeder layer, C. Monoclone in the highly proliferated form (magnification: ×20), and D. Monoclone in the growing form (magnification: ×40).

**Table 1:** ELISA mouse monoclonal antibody (mAb) isotyping
Class and subclass monoclonal antibodies of the 3-D5


Class	IgG1	IgG2a	IgG2b	IgG3	IgA	IgM	Kappa	Lambda

Clone3-D5	1.021	0.156	0.154	0.109	0.157	0.131	1.632	0.144


In addition, the purified antibody showed immunoreactivity with the immunizing peptide in ELISA. Western blotting technique was performed to see the pattern of reactivity of anti- CD34 mAbs with different cell lines such as Raji, HL-60, and umbilical cord blood (UCB)- derived CD34 cells. Only one specific band was visualized in 110 kDa in the UCB lysate (Fig 3B) .There was good concordance between the results obtained in both the ELISA and Western blot assays. Taken together, these results illustrated that this antibody was highly specific and functional in applications such as ELISA and Western blot assays.

**Fig 3:**
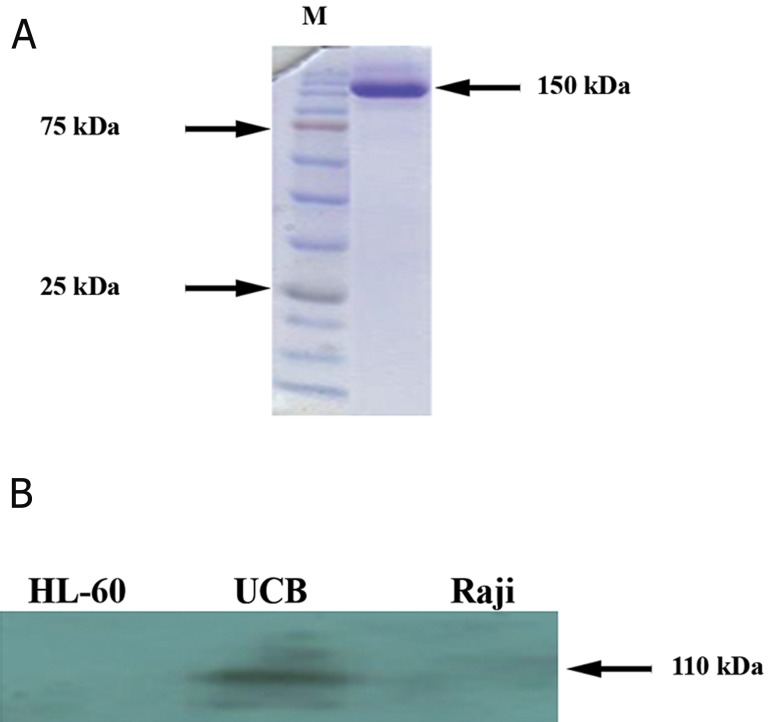
SDS-PAGE of monoclonal antibodies (mAbs) isolated by protein-A-sepharose chromatography A. In SDS-PAGE (non-reducing condition) only one 150 kDa (molecular weight of IgG) band appeared that demonstrated the purified antibody and Western blotting analysis B. Only one specific band was seen at 110 kDa (molecular weight of CD34) in UCB-derived CD34 cell lysate.

Here, we have employed a peptide-based antibody generation protocol for producing antibody against human CD34 using a new immunization strategy. The use of synthetic peptides as immunogens is generally applied when either the complete protein is not available in sufficient quantities to carry out an adequate immunization protocol or to obtain antibodies that have the capability to recognize only specific regions of a polypeptide chain (11). Synthetic peptides offer the opportunity for a very fast shortcut to overcome a lack of protein. The secondary and tertiary configuration of the peptides, their length, hydrophilicity and location in the native molecule may all be important factors in generating useful antibodies (12). One problem with utilizing a peptide-based antigen is that, because of their small size, peptides are not likely to elicit a robust stimulation of the immune system. KLH has been shown to be an effective carrier protein for immunization with short peptides in the high-yield production of antibodies for research, biotechnology and therapeutic applications (13).

Until now, a large series of CD34-specific mAbs have been developed. Civin et al. produced and developed high affinity murine monoclonal antibodies (My10) that recognized CD34 with a high affinity for diagnosis of hematopoietic progenitor cell. KG-1a cells that possess a large amount of CD34 have been used for immunization (14). The QBEnd antibody, which belongs to class II CD34 epitope mAbs, has been used to isolate HSCs (15).

In conclusion, anti-CD34 mAb can be used in the diagnosis of hematologic malignancies, solid tumors, and immunodeficiency diseases, isolation of hematopoietic progenitor cells, disease monitoring, and *in vitro* differentiation studies. Anti- CD34 mAb may represent a powerful tool for the positive selection or depletion of cells that express human CD34 antigen. Upon our findings, it can be proposed that this particular approach for production of an anti-CD34 peptide antibody is feasible and cost-effective. This study clearly indicates that the produced antibodies can be used in research and diagnosis as well as clinical applications if produced in the chimeric form.
